# Clinical and Physiological Significance of F-Wave in Spinocerebellar Ataxia Type 3

**DOI:** 10.3389/fneur.2020.571341

**Published:** 2020-09-29

**Authors:** Qiong Cai, Wenxiao Xu, Songjie Liao, Yinxing Liang, Chao Wu, Xunhua Li

**Affiliations:** ^1^Department of Neurology, The First Affiliated Hospital, Sun Yat-sen University, Guangzhou, China; ^2^Guangdong Provincial Key Laboratory of Diagnosis and Treatment of Major Neurological Diseases, National Key Clinical Department and Key Discipline of Neurology, Guangdong Provincial Engineering Center for Major Neurological Disease Treatment, Guangdong Provincial Translational Medicine Innovation Platform for Diagnosis and Treatment of Major Neurological Disease, Guangdong Provincial Clinical Research Center for Neurological Diseases, Guangzhou, China

**Keywords:** spinocerebellar ataxia type 3, F-wave amplitude, giant F-wave, F/M amplitude ratio, scale for the assessment and rating of ataxia

## Abstract

**Objective:** To evaluate the characteristics of F-wave in spinocerebellar ataxia type 3 (SCA3) patients and preclinical carriers of SCA3 gene mutation (PreSCA3), and explore the relationship between disease severity and F-wave parameters and evaluate F-wave parameters as potential biomarkers for monitoring of disease progression in SCA3.

**Methods:** We performed F-wave recordings in median, ulnar and tibial nerves of 39 SCA3 patients, 20 PreSCA3, and 27 healthy controls, and compared F-wave parameters between them.

**Results:** In all nerves studied, the mean F-wave amplitude, maximum F-wave amplitude, and F/M amplitude ratio were significantly increased in the SCA3 patients in comparison with the normal controls. And the minimal F-wave latency of SCA3 patients was significantly prolonged and the F-wave persistence (%) was significantly decreased in the median nerve. For the PreSCA3, the maximum F-wave amplitude was significantly higher than normal controls for both median, ulnar, and tibial nerves. The mean F-wave amplitude and F/M amplitude ratio in all nerves were comparable between PreSCA3 and normal controls. The frequency of giant F-wave and frequency of patients with giant F-wave were similar between PreSCA3 and SCA3. The values of F/M amplitude ratio in both median, ulnar, and tibial nerves were correlated positively with disease severity and disease duration.

**Conclusion:** Significant F-wave abnormalities occur in patients with SCA3, even in PreSCA3. F-wave may therefore reveal subclinical alterations and provide objective parameters for evaluating the progression of SCA3.

## Introduction

Spinocerebellar ataxia (SCA) type 3 is caused by cytosine-adenine-guanine (CAG) repeat expansions in *ATXN3* gene, which represents the most prevalent subtype of SCAs in China (1). Although gait ataxia is the usual chief complaint, other distinguishing clinical features include eye movement abnormalities (2), pyramidal signs, peripheral neuropathy, and extrapyramidal manifestations (3). Previous studies have demonstrated that peripheral neuropathy was highly detected in SCA3 and might be an important source of disability (4, 5). In some patients, motor axons were specifically damaged in a pattern resembling motor neuron disease (6).

F-wave, as the late response following peripheral electrical stimulation of the motor fibers, results from backfiring of antidromically activated motor neurons, which could effectively supplements the conventional nerve conduction studies (7). F-wave latency has been proven as valuable marker of conduction property of motor axons. Decreased F-wave amplitude and persistence, prolonged F-wave latency, and F-wave chronodispersion were certified as signs of damage in the lower motor neuron (8, 9).

F-wave has also been proposed as indicator of upper motor neuron dysfunction by reflecting changes in the excitability of motor neuron, although this was less commonly noted (10). Increased F-wave amplitude and increased F/M amplitude ratio have been previously reported as typical electrophysiological features in patients with pure upper motor neuron diseases, which is attributed to the imbalance of excitatory and inhibitory postsynaptic potentials on spinal motor neurons (8).

There was a previous study investigating F-wave persistence and latency between SCA3 patients with and without cramps, the study found no difference between them (11). Additionally, another study of 12 SCA3 patients found that increased mean F-wave amplitude in ulnar nerve, but no correlations were found between F-wave amplitude and clinical parameters (12). However, other variable characteristics of F-wave reflecting both upper and lower motor neuron properties in symptomatic SCA3 have not been explored up till now. Could these changes already be detected in preclinical carriers of SCA3 mutation (PreSCA3) (13) also have not been explored up. In particular, the difference of F-wave parameters between SCA3 patients and PreSCA3 has not been illuminated.

Therefore, we aimed to record distinguishing F-wave changes in SCA3, especially in PreSCA3. And to explore the relationship between disease severity, duration, and F-wave characteristics, with the purpose of evaluating F-wave parameters as potential biomarkers for monitoring disease progression in SCA3.

## Materials and Methods

### Participants

A total of 39 SCA3 patients and 20 PreSCA3 were recruited in the Neurogenetic outpatient clinic of The First Affiliated Hospital of Sun Yat-sen University, Guangdong, China, from January 2018 to January 2020. And 27 healthy volunteers served as controls. Participants with diabetes mellitus, alcohol abuse, other systemic disorders, or peripheral neurological diseases had been excluded. All participants underwent a clinical and electrophysiological examination. To measure the severity of ataxia, we used the Scale for the Assessment and Rating of Ataxia (SARA) that has been validated (14). Ethics approval was obtained by the ethics board of the Ethics Committee of the First Affiliated Hospital of Sun Yat-sen University, Guangdong, China [No. (2014) 23]. The study was carried out only after written informed consent was obtained from all participants.

### Motor Nerve Conduction Study

Electromyography (Keypoint; Medronic, Skovlunde, Denmark) was used for all neurophysiological studies. All subjects were examined in a supine position as relaxed as possible to avoid voluntary activity, and with the skin temperature remained 32°C or warmer during the experiment. Standard motor nerve conduction studies were conducted as described elsewhere (15). Motor nerve conduction comprised the median, ulnar, tibial, and peroneal nerves. The active electrode was placed over the middle of the muscle belly (abductor pollicis brevis, abductor digiti minimi, abductor halluces brevis, and extensor digitorum brevis, respectively) and the reference electrode was placed on the tendon distally. Stimulation was performed at the wrist (7 cm from the recording electrode) and elbow for the median and ulnar nerves; at the ankle and knee for the tibial and peroneal nerves. Another surface electrode was placed between the recording and stimulating electrodes served as a ground. Motor nerve conduction parameters, including distal motor latency (DML) and peak-to-peak compound muscle action potential (CMAP) amplitude, were analyzed.

### F-Wave Study

We used the F-wave program installed in the EMG machine with the filter range of 20 Hz−3 kHz, amplifier gain setting at 0.5 mV for F-waves, and 5 mV for M responses and the sweep speed at 5 ms/div for upper limbs and 10 ms/div for lower limbs. Forty consecutive supramaximal stimuli were delivered in median nerve, ulnar nerve, and tibial nerve of all subjects with a frequency of 1 Hz and duration of 0.1 ms. The supramaximal intensity defined as 20% greater than the maximal stimulus, which just activated the entire group of axons, thus elicting a maximal amplitude CMAP. The A-wave were excluded from the F-wave measures and were defined as identical late responses in more than 8 of 20 traces with a constant latency (16). The minimum amplitude (peak-to-peak) used to identify the F-wave was 40 μV (17). The following parameters were analyzed: minimal F-waves latency, mean and maximal amplitude, the percentage mean of F-wave amplitude/CMAP amplitude ratio (F/M amplitude ratio), F-waves persistence (percent of definable traces with any F responses in a series of 40 stimuli), frequency of giant F-wave, and frequency of patients with giant F-wave (8). A large amplitude F-wave exceeding the mean value plus 2 standard deviations (SD) of the largest F-wave recorded in healthy subjects was considered a giant F-wave (18). In the present study, we defined the amplitude for a giant F-wave according the previous study (19), which was 1473.80 mV in the median nerve, 1,293.69 mV in the ulnar nerve, and 1,045.46 mV in the tibial nerve.

### Statistical Analyses

Descriptive statistics were given as mean and SD. The Kolmogorov–Smirnov test was used to estimate the normality of the data. The homogeneity was tested using a Levene test. If the data were normally distributed, a univariate ANOVA was performed for three-group comparisons with a *post-hoc* Newman–Keuls test. For data that were not normally distributed, nonparametric statistical comparisons were performed. The Kruskal–Wallis *H* test with a *post-hoc* Mann–Whitney *U-*test with Bonferroni correction was used. A χ^2^ test was used for categorical data.The relationship between two variables was assessed by Pearsons correlation coefficient. Statistical significance was set at *P* < 0.05. All statistical analyses were conducted using SPSS for windows, version 20 (SPSS, Inc., Chicago, Illinois, USA).

## Results

### Clinical Profiles

The clinical characteristics of the participants are summarized in Table 1. The CAG repeat size was comparable between SCA3 and PreSCA3. The mean age did not differ significantly between groups, with the exception of the PreSCA3 group, where it was younger than in the control and SCA3 group (*P* < 0.001, 0.001). And the height was comparable among groups. The SARA score of the SCA3 patients in the present study was 13.39 ± 6.17 (range 5.00–34.50), and the disease duration at the time of examination was 6.36 ± 3.82 years (range 1.00–20.00 years). The presence of hyperreflexia was found in 79.5% of SCA3 and 5% of PreSCA3. However, the presence of hyporeflexia was seen in 26.5% of SCA3 and 5% of PreSCA3. Impaired vibration sense was rare in SCA3 group.

**Table 1:** Clinical profiles of the participants in the study.

**Parameters**	**SCA3(1)**	**PreSCA3(2)**	**Control (3)**	***p*****-value**		
				**1 vs. 3**	**2 vs. 3**	**1 vs. 2**
No.	39	20	27			
Age, year (range)	41.46 ± 10.69 (23–65)	29.00 ± 8.58 (18–51)	40.15 ± 10.74 (22–63)	>0.05	**<0.001**	**<0.001**
Height (cm)	161.5 ± 8.06	164.4 ± 5.08	161.8 ± 7.41	>0.05	>0.05	>0.05
Duration, year	6.36 ± 3.82 (1–20)	0.10 ± 0.31	NA	NA	NA	**<0.001**
CAG	75.33 ± 3.79 (67–84)	74.20 ± 2.90 (67–79)	NA	NA	NA	0.255
SARA score	13.39 ± 6.17 (5–34.5)	0.50 ± 0.69 (0–2)	NA	NA	NA	**<0.001**
Hyperreflexia in lower limbs (%)	79.5	5.0	0	NA	NA	**<0.001**
Hyporeflexia in lower limbs (%)	25.6	5.0	0	NA	NA	0.054
Extensor plantar (%)	59.0	0	0	NA	NA	NA
Impaired vibration sense (%)	5.1	0	0	NA	NA	NA

*SCA3, spinocerebellar ataxia type 3; PreSCA3, preclinical carriers of SCA3 gene mutation; NA, not applicable. Values are mean ± SD. Values with significant differences printed in bold characters*.

### CMAP Parameters

The results of nerve conduction studies of the SCA3 patients, PreSCA3, and the normal controls are shown in Table 2.

**Table 2:** Results of nerve conduction studies.

	**SCA3(1)**	**PreSCA3(2)**	**Control (3)**	***p*****-value**			**Overall**
				**1 vs. 3**	**2 vs. 3**	**1 vs. 2**	***P*-value**
**DML (ms)**							
Median nerve	3.23 ± 0.50	2.81 ± 0.25	2.98 ± 0.34	**0.032**	0.216	**0.001**	**0.002**
Ulnar nerve	2.38 ± 0.44	2.14 ± 0.25	2.29 ± 0.30	0.375	0.188	0.08	0.072
Tibial nerve	3.61 ± 0.66	3.26 ± 0.61	3.59 ± 0.64	0.860	0.064	0.06	0.114
Peroneal nerve	3.71 ± 0.76	3.48 ± 0.51	3.20 ± 0.48	**0.004**	0.177	0.349	**0.017**
**CMAP amplitude (mV)**							
Median nerve	13.59 ± 3.12	16.72 ± 3.68	15.34 ± 2.96	0.120	0.211	**0.013**	**0.031**
Ulnar nerve	13.83 ± 3.16	13.69 ± 2.85	15.20 ± 2.85	0.617	0.260	0.406	0.527
Tibial nerve	20.08 ± 8.55	21.42 ± 7.25	22.02 ± 8.21	0.126	0.115	0.570	0.175
Peroneal nerve	6.18 ± 2.89	9.00 ± 3.86	8.82 ± 3.17	**0.003**	0.832	0.059	**0.007**

*SCA3, spinocerebellar ataxia type 3; PreSCA3, preclinical carriers of the SCA3 gene mutation; DML, distal motor latency; CMAP, compound muscle action potential; NA, not applicable. Values are mean ± SD. Values with significant differences printed in bold characters*.

The DMLs and CMAP amplitude in the tibial and ulnar nerves were comparable between SCA3 patients, PreSCA3, and controls. The DML and CMAP amplitude were comparable between SCA3 patients and PreSCA3, except for the significantly prolonged DML and decreased CMAP amplitude in the median nerves of the SCA3 patients (*P* < 0.05). When compared with the controls, the CMAP amplitude of SCA3 patients was significantly decreased in peroneal nerves and the DML was significantly prolonged in both median and peroneal nerves (all *P* < 0.05).

It should be noted, however, that all of the patients had CMAP values within the normal range of our laboratory.

### F-Wave Findings in PreSCA3 and SCA3

Clear F-wave recordings were obtained from all participants in our study. Similar to previous studies (15, 20), there were no correlations between age and F-wave amplitude or F-wave latency. Table 3 shows the results of F-wave studies performed in the nerves of upper and lower extremity.

**Table 3:** F-wave studies in upper and lower extremity nerves.

**Parameters**	**SCA3 (1)**	**PreSCA3 (2)**	**Control (3)**	***p*****-value**			**Overall**
				**1 vs. 3**	**2 vs. 3**	**1 vs. 2**	***P*-value**
**Median nerve**							
Min F latency (ms)	25.16 ± 1.83	24.08 ± 1.76	24.09 ± 1.53	**0.032**	0.996	**0.040**	**0.040**
F persistence (%)	89.92 ± 18.96	97.99 ± 6.23	99.54 ± 1.96	**0.021**	0.853	**0.045**	**0.032**
Mean Famp (μV)	332.2 ± 165.4	293.6 ± 113.6	241.3 ± 62.92	**0.008**	0.150	0.320	**0.030**
Maximum Famp (μV)	769.6 ± 405.6	733.6 ± 296.7	485.9 ± 186.0	**0.001**	**0.014**	0.711	**0.004**
F/M amplitude ratio (%)	2.48 ± 1.06	1.79 ± 0.72	1.67 ± 0.60	**0.001**	0.543	**0.009**	**0.001**
Giant F-waves (%)	0.66 ± 3.44	0 ± 0	0 ± 0	NA	NA	NA	NA
Frequency of Patients with giant F-waves	2/34	0/18	0/27	NA	NA	NA	NA
**Ulnar nerve**							
Min F latency (ms)	24.53 ± 1.96	23.93 ± 1.26	23.91 ± 1.57	0.214	0.968	0.239	0.342
F persistence (%)	99.61 ± 1.25	97.43 ± 14.15	100 ± 0	NA	NA	0.735	NA
Mean Famp (μV)	369.3 ± 135.8	316.9 ± 140	263.3 ± 70.72	**0.007**	0.221	0.174	**0.024**
Maximum Famp (μV)	879.5 ± 457.2	712.8 ± 400.8	468.7 ± 124.9	**0.001**	**0.048**	0.192	**0.003**
F/M amplitude ratio (%)	2.96 ± 1.62	2.34 ± 0.91	1.83 ± 0.56	**0.003**	0.229	0.100	**0.011**
Giant F-waves (%)	1.18 ± 2.84	0.53 ± 1.78	0 ± 0	NA	NA	0.371	NA
Frequency of Patients with giant F-waves	6/34	2/19	0/27	NA	NA	0.487	NA
**Tibial nerve**							
Min F latency (ms)	43.15 ± 3.54	41.99 ± 2.27	43.28 ± 2.89	0.871	0.174	0.194	0.337
F persistence (%)	100 ± 0	100 ± 0	100 ± 0	NA	NA	NA	NA
Mean Famp (μV)	553.5 ± 271.6	484.3 ± 287	400.8 ± 157.2	**0.012**	0.230	0.317	**0.043**
Maximum Famp (μV)	1020 ± 456.6	1074 ± 658.4	687.5 ± 321.8	**0.006**	**0.007**	0.687	**0.008**
F/M amplitude ratio (%)	2.96 ± 1.35	2.33 ± 1.13	2.07 ± 1.32	**0.006**	0.462	0.086	**0.018**
Giant F-waves (%)	13.41 ± 19.28	9.10 ± 21.32	0.91 ± 2.94	**0.004**	**0.012**	>0.05	**0.015**
Frequency of Patients with giant F-waves	19/38	10/19	3/27	**0.001**	**0.002**	0.851	**0.002**

*SCA3, spinocerebellar ataxia type 3; PreSCA3, preclinical carriers of the SCA3 gene mutation; NA, not applicable. Values are mean ± SD. Values with significant differences printed in bold characters*.

When compared with the controls, the maximum F-wave amplitude of PreSCA3 was significantly increased in the median, ulnar and tibial nerves (all *P* < 0.05). And the minimal F-wave latency, F-wave persistence, as well as the mean F-wave amplitude and F/M amplitude ratio were comparable among PreSCA3 and healthy controls. For the SCA3, the minimal F-wave latency was significantly prolonged and the F-wave persistence (%) was significantly decreased in the median nerves compared to controls (*P* < 0.05, respectively). There were no significant differences between SCA3 patients and healthy controls for the minimal F-wave latency and F-wave persistence of the ulnar and tibial nerves. In all nerves studied, the mean F-wave amplitude, maximum F-wave amplitude, and F/M amplitude ratio were significantly increased in SCA3 in comparison with controls (all *P* < 0.05). However, the minimal F-wave latency and F/M amplitude ratio were significantly increased of SCA3 and F-wave persistence was significantly lower in the median nerves than in the PreSCA3. The F-wave parameters from the ulnar and tibial nerves were not significantly different between SCA3 and PreSCA3.

**Figure 1:**
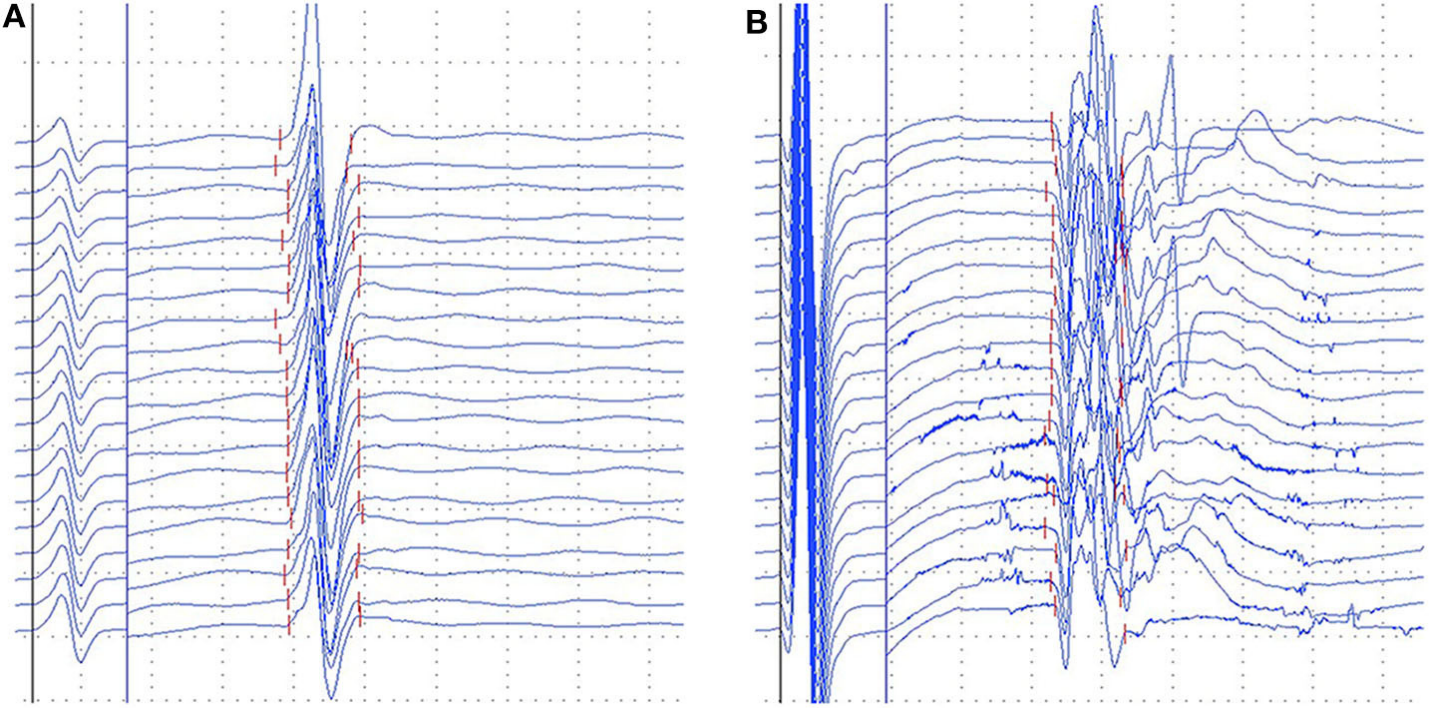
Representative images of gaint F-waves in the preclinical carriers and SCA3 patients. **(A)** gaint F-waves of tibial nerve in the preclinical carriers with 92.1% persistence; **(B)** gaint F-waves of tibial nerve in the SCA3 patients with 67.5% persistence. Dural gain was used; during the M-wave, the gain was 5 mV/div, and during the F-wave, the gain was 0.5 mV/div.

Giant F-wave occurred more frequently in the tibial nerves than the median and ulnar nerves in both PreSCA3 and SCA3. Figure 1 presents representative examples of giant F-wave traces for the tibial nerves recorded from PreSCA3 and SCA3 patients. Compared to 50% of SCA3 patients showing giant F-wave in at least one nerve, 11.1% of controls presented with giant F-wave (*P* < 0.05). The frequency of patients with giant F-wave was similar between SCA3 and PreSCA3 (*P* > 0.05).

### Correlations of F-Wave Findings

**Figure 2:**
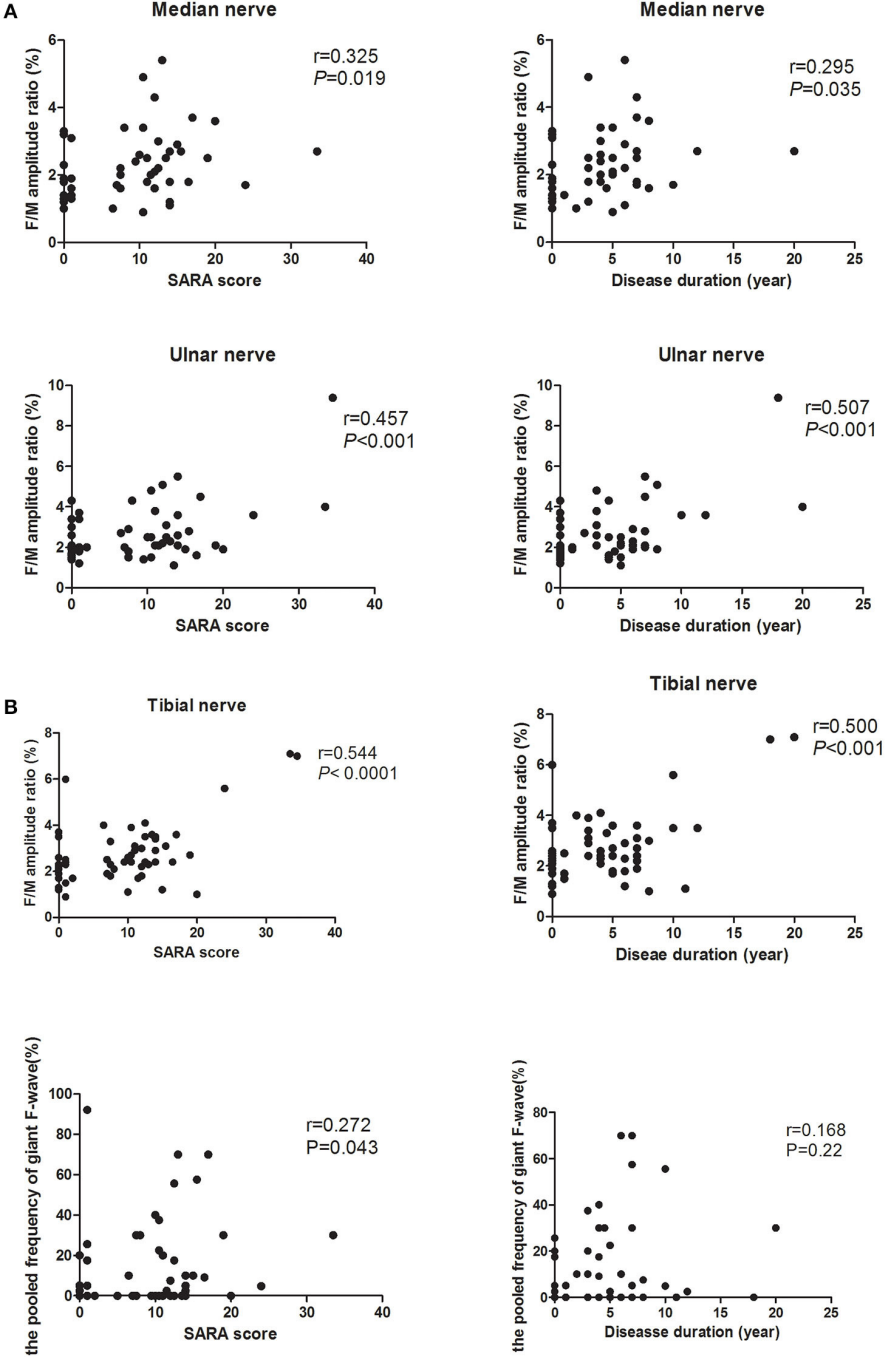
Correlation of F-wave parameters between the disease severity and disease duration. **(A)** Correlation of F/M amplitude ratio in both median, ulnar, and tibial nerves between the disease severity and disease duration. It shows that the higher SARA score and the longer disease duration in a recording, the higher the F/M amplitude ratio. **(B)** Correlation between the pooled frequency of giant F-wave and the disease severity or disease duration.

The possible relations of F-wave parameters and clinical variables for both PreSCA3 and SCA3 patients were performed. The values of F/M amplitude ratio in tibial, median, and ulnar nerves correlated positively with the severity of the disease as determined by the SARA score (*r* = 0.544, *P* < 0.0001; *r* = 325, *P* < 0.05; *r* = 0.457, *P* < 0.001, respectively) and the disease duration before electrophysiological examinations (*r* = 0.500, *P* < 0.001; *r* = 0.295, *P* < 0.05; *r* = 0.507, *P* < 0.001, respectively; Figure 2A). No significant relationship was observed between the mean F-wave amplitude in all studied nerves and the SARA score or the disease duration. Likewise, there were no correlations between the maximum F-wave amplitude and the SARA score or the disease duration. Among SCA3 patients and PreSCA3, the pooled frequency of giant F-wave (13.48 ± 21.08%, range 0–92.1%) correlated significantly with the disease severity (*r* = 0.272, *P* = 0.043) but not with disease duration (*r* = 0.157, *P* = 0.247; Figure 2B).

## Discussion

In this cohort, we compared the F-wave parameters between SCA3 patients, PreSCA3, and normal controls. In SCA3, these F-wave features included mildly prolonged F-wave latency and decreased F-wave persistence, which may attributed to lower motor neuron involvement, increased F-wave amplitude, and increased F/M amplitude ratio which attributed to upper motor neuron involvement, when compared to the healthy controls. And in PreSCA3, we observed increased maximum F-wave amplitude and increased frequency of giant F-wave as compared to controls (Table 4).

**Table 4:** F-wave characteristics in SCA3 and PreSCA3.

	**Upper motor neuron**	**Lower motor neuron**
	**involvement**	**involvement**
SCA3	Increased mean Famp	Prolonged minimal F latency
	Increased maximum Famp	Decreased F persisitence
	Increased F/M amplitude ratio	
PreSCA3	Increased maximum	/
	Famp	

*SCA3, spinocerebellar ataxia type 3; PreSCA3, preclinical carriers; Famp, F-wave amplitude*.

Our findings are in accordance with previous reports which showed increased mean F-wave amplitude in ulnar nerves (12). A strength of our study is that we also investigated the F-waves characteristics in PreSCA3. The significant increased maximum amplitude of F-wave observed in PreSCA3 could be potentially attributed to imbalance of central impulses in favor of excitatory, resulting in enhanced anterior horn cells excitability (21). This alteration suggests that the impairment of upper motor neurons seems to occur before the cerebellar syndrome appears in PreSCA3.

The CMAP amplitude are useful parameters for evaluating motor neuron loss, whereas the F-wave may be a direct probe of dysfunction or instability in upper motor neurons disorder (22). These results indicate that this estimate probably reflects the significance of F-wave parameters in SCA3. A significant reduction of median nerve F-wave persistence compared to PreSCA3 and controls was observed in SCA3 patients. The low mean value of F-wave persistence in SCA3 patients may relate to loss of fast conduction neurons, which indicated lower motor neuron damage (23, 24). The amplitude of F-wave are indice of motor unit size and motoneuron excitability (25). Upper motor neurons damage has been shown to increase the amplitude of F-wave (7). Previous research demonstrated that the increased F-wave amplitude in amyotrophic lateral sclerosis generation by enlarged, reinnervated motor units, which were synchronized due to the pyramidal lesion (23). We hypothesized that increased F-wave amplitude in SCA3 had a similar pathogenesis. Furthermore, previous study showed involvement of the corticospinal tract in 60 to 67% of SCA3 patients who showed pyramidal signs (26, 27). Features suggestive of pyramidal tract dysfunction have been documented in PreSCA3 as well (13). In our study, the presence of hyperreflexia was found in 79.5% of SCA3 and 5% in PreSCA3. Moreover, we have also analyzed the F/M amplitude ratio, which is significantly less influenced by muscle wasting and found that it markedly increased in all examined nerves of SCA3. The F/M amplitude ratio quantifies the proportion of the motoneuron pool that is activated during a series of F-waves (28). The significantly increased F/M amplitude ratio in SCA3 patients compared to PreSCA3 and normal controls, probably caused by upper motor neuron involvement resulting in either increased tendency of the unaffected neurons to generate F-wave or to synchronization of centrifugal impulses (21). The generation of giant F-wave of patients with anterior horn cell disorders may be attributed to axonal sprouting and subsequent reorganization of a muscle's innervation (18, 19). The significant increase in the number of giant F-wave in tibial nerves of SCA3 patients and PreSCA3 may imply the preferential affection of spinal motoneurons innervating the abductor halluces brevis. Interestingly, some of these abnormalities appeared during the preclinical stage.

The results of the motor nerve conduction study revealed that the peroneal nerve CMAP amplitude was decreased and the DML was prolonged in SCA3 patients as compared to controls. This could be attributed to peripheral nerve axonal degeneration and loss of the fastest conducting fibers (4, 29). And this suggested that the lower limbs were more vulnerable. In our preliminary study, we also observed predominantly reduced amplitudes of sensory nerve action potential (SNAP) in most SCA3 patients. These findings are in agreement with the literature that SCA3 patients revealed sensorimotor neuropathy (5).

We have observed a positive correlation between F/M amplitude ratio of median, ulnar and tibial nerves and disease duration. And F/M amplitude ratio also correlated with progression of SARA scores. These findings raise the possibility that F/M amplitude ratio may be markers of disease progression over time. Prospective studies with larger samples and longer follow-up periods will help to better evaluate this issue.

## Conclusion

Based on the findings of the present study, the characteristic features of F-wave in SCA3 were a significant increase F-wave minimal latency, F-wave amplitude, F/M amplitude ratio, and increased frequency of giant F-wave, and a decrease of F-wave persistence. Meanwhile, a significant increase F-wave maximum amplitude and frequency of giant F-wave were also observed in PreSCA3. F-waves may therefore reveal subclinical alterations and provide objective parameters for evaluating the progression of SCA3.

## Data Availability Statement

The raw data supporting the conclusions of this article will be made available by the authors, without undue reservation.

## Ethics Statement

The studies involving human participants were reviewed and approved by Ethics Committee of the First Affiliated Hospital of Sun Yat-sen University, Guangdong, China. The patients/participants provided their written informed consent to participate in this study.

## Author Contributions

The authors declare that they have each made substantial contributions to the conception, acquisition, analysis, and interpretation of the manuscript. All authors have critically revised the manuscript for intellectual content and have given their approval for the final version to be published.

## Conflict of Interest

The authors declare that the research was conducted in the absence of any commercial or financial relationships that could be construed as a potential conflict of interest.
